# Source-credibility information and social norms improve truth discernment and reduce engagement with misinformation online

**DOI:** 10.1038/s41598-024-57560-7

**Published:** 2024-03-22

**Authors:** Toby Prike, Lucy H. Butler, Ullrich K. H. Ecker

**Affiliations:** 1 School of Psychological Science, University of Western Australia, Perth, Australia; 2 School of Psychology, University of Adelaide, Adelaide, Australia

**Keywords:** Human behaviour, Social behaviour

## Abstract

Misinformation on social media is a pervasive challenge. In this study (*N* = 415) a social-media simulation was used to test two potential interventions for countering misinformation: a credibility badge and a social norm. The credibility badge was implemented by associating accounts, including participants’, with a credibility score. Participants’ credibility score was dynamically updated depending on their engagement with true and false posts. To implement the social-norm intervention, participants were provided with both a descriptive norm (i.e., most people do not share misinformation) and an injunctive norm (i.e., sharing misinformation is the wrong thing to do). Both interventions were effective. The social-norm intervention led to reduced belief in false claims and improved discrimination between true and false claims. It also had some positive impact on social-media engagement, although some effects were not robust to alternative analysis specifications. The presence of credibility badges led to greater belief in true claims, lower belief in false claims, and improved discrimination. The credibility-badge intervention also had robust positive impacts on social-media engagement, leading to increased flagging and decreased liking and sharing of false posts. Cumulatively, the results suggest that both interventions have potential to combat misinformation and improve the social-media information landscape.

## Source-credibility information and social norms improve truth discernment and reduce engagement with misinformation online

The proliferation of misinformation on online social-media platforms is an issue of contemporary concern^1–3^. A substantial amount of research has thus explored ways to reduce the formation and maintenance of misinformation-driven false beliefs, and the dissemination of misinformation [^4–11^; for reviews, see^12,13^]. In this study, we used a realistic social-media simulation to explore the impact of two potentially scalable interventions to reduce people’s inclination to share dubious content: source-credibility information and the provision of a social norm. The common feature of these interventions is that they use the desire to avoid reputational damage to motivate better engagement with information. In the case of source-credibility information, people may avoid sharing misinformation because doing so could lead to a reduction in their perceived credibility. In the case of a social norm against spreading misinformation, people may be motivated to not share misinformation because there is a desire to avoid the reputational harm that comes from violating norms.

The first factor examined in the present study is source-credibility information. It is well-known that the credibility of a source can influence perceptions of a message^14–17^, including misleading messages^18–20^ and misinformation corrections^21–23^. However, this body of research has generally examined the role of source credibility by providing specific information about why (or why not) the source should be trusted, for example by comparing the effect of information from a real news source and a made-up news source^19^ or by manipulating whether the source has a potential conflict of interest^21^. There is less research into how people interpret source-credibility information that is presented as a standalone rating. Kim et al.^24^ examined the impact of a standalone numerical source-credibility rating on belief in articles, with mixed results, finding a significant effect on belief in only one of the two experiments. Additionally, the study found that credibility ratings did not impact liking, sharing, or commenting on the articles. However, unlike news articles, for which people may generally have pre-existing beliefs about the credibility of sources, on social-media platforms people regularly encounter information from unknown sources. Therefore, given the difficulty of gauging source credibility on social media, it may be an environment in which source-credibility information is particularly salient and influential.

A potentially even more influential facet of source-credibility information, which has yet to be examined, is how people respond when their own credibility is being monitored and updated in response to their online behavior. In addition to the important role that credibility plays in persuading others, credibility also influences the evaluation of others when deciding who to interact with^25^. In a social-media environment, there is thus a risk that sharing false information can lead to reputational damage that decreases others’ willingness to engage^26^. Currently, the risk involved in sharing or otherwise positively interacting with misinformation in social-media environments is low because the only way that reputational damage can be incurred is through fact-checkers or other users directly engaging and disputing or correcting the misinformation. Additionally, even if disputations or corrections do occur, they may be limited to one specific post or comment thread, meaning that other users may remain unaware that the account had spread misinformation. However, by directly linking a credibility rating to an account, and making it visible to other users, the risk that spreading misinformation will lead to reputational damage is greatly increased. In such an environment, social-media users should therefore be motivated to avoid actions that could substantively reduce their perceived credibility online, and should thus reduce their sharing of misinformation^24,26,27^.

A second factor investigated in the present study is the provision of a social norm. Social norms are known to have a measurable impact on people’s attitudes and behaviors^28–30^. Kaplan and Miller^31^ argued that social-norm impacts can be due to informational influence (i.e., norm information provides potentially persuasive evidence about the world) or normative influence (i.e., people fear social exclusion resulting from norm violations). In the misinformation realm, the normative signal associated with the endorsement of social-media messages has been found to influence false-message belief both before and after correction^32–34^, and a social-norming intervention has been found to reduce belief in equivocal claims^35^.

As far as interactions with misinformation on social media are concerned, there is some tentative evidence that the provision of social norms may improve social-media engagement. Specifically, Andı and Akesson^36^ and Gimpel et al.^37^ found that presenting a social norm led to reduced sharing and increased flagging of false news articles, respectively. However, efficacy seems to depend on the type of social-norm information presented: Gimpel et al.^37^ found that presenting a descriptive norm alone (i.e., without an injunctive norm) did not significantly increase flagging of misinformation. Similarly, Epstein et al.^38^ found a descriptive-norm intervention was ineffective in isolation but effective when combined with digital-literacy tips or a question asking how important it was to only share accurate information. In general, an important limitation of social-norm interventions in this context is that even if presenting a social norm increases participants’ intention to improve their engagement with true and false information, the intervention’s effectiveness may be limited by a person’s ability to distinguish between true and false posts. Thus, to be fully effective social-norm interventions may benefit from the labeling of false or misleading content^39^.

### The present study

The present study simulated a social-media network in an experimental survey, using a social-media simulation^40^. Participants were presented with social-media posts containing either true or false claims, and were asked to engage with the posts (i.e., “like” or “share” them, or “flag” them as misleading) as they would on social media in order to grow their following. Posts were fact-checked (i.e., false claims were refuted; true claims affirmed) in the comment section of each post. Participants’ follower number changed dynamically depending on their engagement with individual posts. The main dependent variable was a composite score of participant engagement with the posts. Claim belief was included as an additional dependent variable, measured at the end of the experiment.

Two factors were manipulated: (1) the presence of source-credibility information, and (2) provision of a social norm against sharing of false information. Regarding (1), in conditions with source-credibility information, all post sources and the participant had a credibility score. This means that the profiles of both the participant and other virtual network members featured a badge that indicated source credibility, thereby identifying accounts as more or less reliable sources of information. Participants’ credibility was progressively mapped through dynamic, trial-by-trial changes that depended on participants’ engagements with the posts (e.g., liking or sharing a false post tended to reduce credibility, liking or sharing a true post tended to increase credibility). The credibility score of virtual sources did not change because each source was only associated with one post. No explicit instructions regarding credibility were given; however, it is reasonable to assume that presence of source-credibility information would implicitly alter participants’ motivations. Thus, the participants’ aim in these conditions was arguably to achieve a growing number of followers while maintaining some self-determined level of acceptable credibility. Regarding (2), in social-norm conditions, instructions provided a social norm against the spreading of falsehoods, comprising both a descriptive norm (i.e., that most people do not share misinformation) and an injunctive norm (i.e., that sharing misinformation is the wrong thing to do). It is known that the combination of descriptive and injunctive elements tends to achieve greater efficacy than either element in isolation^29,41,42^.

Consistent with prior research, we expected the social norm to improve engagement behavior (specifically, less liking/sharing, and more flagging of false posts), particularly because fact-checks were provided, which enabled participants to discern true from false posts. We also expected that our novel credibility-badge intervention and the dynamic credibility feedback provided to participants would lead to improved engagement behavior. The combination of both interventions was expected to be particularly effective, either due to the norm reducing participants’ willingness to sacrifice credibility to achieve a greater social-media following or the credibility information making norm violations more salient. An additional research question was whether the interventions would reduce belief in false claims, and particularly whether the interventions would improve participants’ ability to discriminate between true and false posts.

## Method

### Participants

An a-priori power analysis suggested a minimum sample size of 100 per cell to detect an effect of *f* = 0.20 (α = 0.05; 1 – β = 0.80) between two conditions. To account for potential exclusions, we recruited 426 adult, U.S.-based participants via Prolific (minimum approval rating: 95%). Participants were excluded based on preregistered exclusion criteria if they (1) self-reported their English language proficiency as only “fair” or “poor” (< 2 on 0–4 scale; *n* = 0); reported that they reside outside of the U.S. (*n* = 0), responded uniformly to the belief questionnaire (> 80% identical ratings; *n* = 1), or suggested that their data should be excluded due to low effort (*n* = 0). Additionally, eleven participants who completed the study reported technical problems (e.g., images not displaying correctly) and were also excluded. Final sample size was thus *N* = 415. This study was approved by the Human Research Ethics Office at the University of Western Australia (reference number: 2019/RA/4/20/6423) and complied with all relevant guidelines and the Declaration of Helsinki. Informed consent was obtained from all participants included in the study.

### Materials

#### Social-media posts

A set of 80 claims was used, 40 of which were objectively true (e.g., “The unicorn is the national animal of Scotland”) and 40 of which were objectively false (e.g., “Most people only use between 10 and 50% of their brains”). Each claim was presented as a social-media post. Each post was associated with a unique account handle (i.e., a source name) and a source icon (the first letter of the handle in a randomly colored circle, similar to a Google-account icon). Each post also featured an image thematically related to the claim. Additionally, information on the number of alleged previous likes, shares, and flags was included. These were determined probabilistically (see Table 1 for parameters of the zero-truncated normal distributions from which numbers were sampled); parameters were identical for true and false posts with the exception that, for the sake of realism, false posts were more likely to have been flagged previously (note that there was a notable difference between parameter and implemented values due to the truncation at zero; descriptive statistics across conditions are provided in Table S1).

**Table 1:** Parameters determining the number of displayed post likes, shares, and flags.

	Likes		Shares		Flags	
	*M*	*SD*	*M*	*SD*	*M*	*SD*
True posts	5	10	1	3	0	0.25
False posts	5	10	1	3	0.5	1

**Figure 1:**
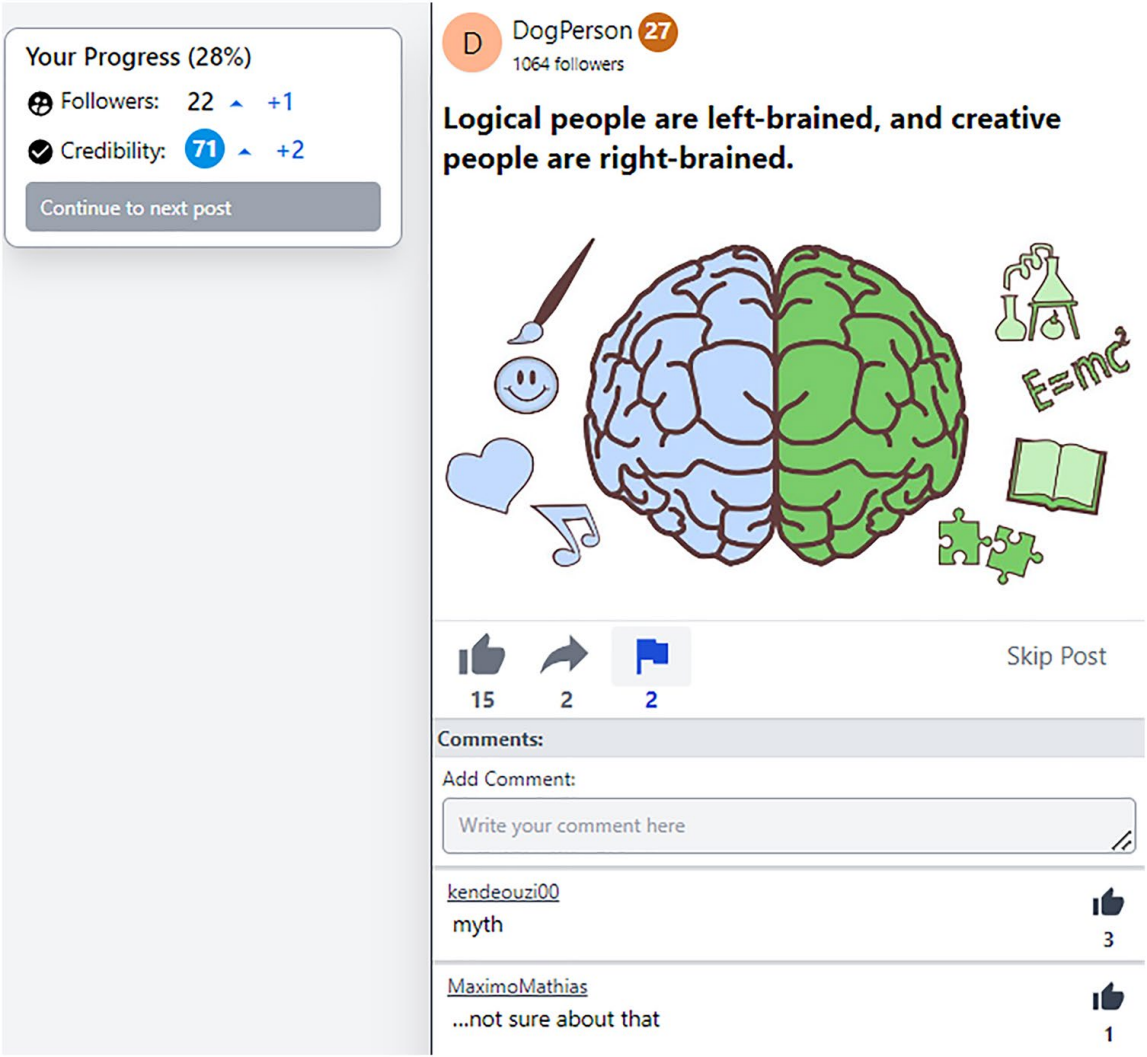
An example false post. The example is from a source-credibility condition (i.e., credibility information is provided for both the post source [27 out of 100] and the participant [71 out of 100]); the participant’s response in this case was to flag the post; information on the dynamic changes (“ + 1” increasing follower number from 21 to 22; “ + 2” increasing credibility from 69 to 71) was only displayed briefly. In the example, the first comment represents a strong refutation; the second comment is a weak refutation. The post image contained within the figure was created by user j4p4n for openclipart.org (https://openclipart.org/detail/336394/brain-hemispheres) and is available under a Creative Commons Zero 1.0 Public Domain License.

Each claim was fact-checked (i.e., corrected or affirmed) via 1–3 comments from other alleged users. Approximately a third of the posts had only one comment; this was a strong fact-check, that is, either a strong refutation (e.g., “MYTH”) or a strong affirmation (e.g., “I know this is true”). A third of posts had two comments, namely a strong fact-check paired with either a weak fact-check (i.e., a weak refutation such as “…not sure about that”, or a weak affirmation such as “Sounds possible”) or a neutral comment, which was typically humorous and thematically related to the post claim without relating to its veracity (e.g., “my ancestors were unicor…I mean Scottish”). A third of posts had three comments, comprising one comment of each type. If multiple fact-check comments were given, they were always compatible (i.e., either both refutational or both affirmative) and fact-checks were valid (i.e., accurate to the best of our knowledge). For posts with multiple comments, the order of comment types was identical across participants, however, roughly counterbalanced across claims. The comments associated with each claim were identical for all participants. The number of comment likes was drawn from normal distribution *N*(*M* = 1, *SD* = 1.5); negative values were replaced with 0, and some minor adjustments were made such that earlier comments (i.e., comments displayed higher up) tended to have more likes and every strong fact-check had at least 1 like to avoid participants interpreting the absence of endorsement as a signal not to trust the fact-check. An example false post is provided in Fig. 1. All posts including comments are available at https://osf.io/whq2v.

Sources had fictional handles; a mixture was used of realistic names (either full names such as “carterrenee” or “DaveChang1997”, or part names such as “RhondaJL” or “anderson_c”), fun names (e.g., “DogPerson”; “pocoloco060”), and meaningless or cryptic handles (e.g., “smhsmhsmh”; “wlihd#”). The same was done with comment sources. Handles were carefully reviewed to ensure the distribution of different handle types was comparable across true and false posts, as well as the different types of comments (i.e., neutral comments as well as weak and strong refutations and affirmations). All handles can be found at https://osf.io/whq2v. Sources’ follower numbers were randomly drawn from *N*(300,500), truncated at 0. Sources’ credibility scores were drawn from *N*(40,30) for false posts and *N*(60,30) for true posts, truncated at 0 and 100. To ensure consistency between conditions, source and post pairings were fixed (implemented source credibility for false claims, *M* = 42.28, *SD* = 22.30; true claims, *M* = 54.93, *SD* = 22.47). This meant that there was a weak relationship between source credibility and veracity of the claims, for the sake of realism.

#### Manipulation information

**Figure 2:**
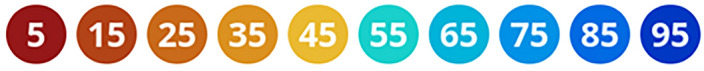
Color-coding of source-credibility badges. Examples represent decile midpoints; actual credibility scores used all integers from 0 to 100.

Regarding the source-credibility intervention, participants received the information that their credibility rating would indicate how credible they are perceived to be on a scale from 0 to 100. Credibility badges were color-coded using the simulator’s default setting (see Fig. 2); scores in the lower deciles were displayed using colors ranging from dark red (0–10) to gold (40–50) and scores in the upper deciles were displayed using colors ranging from turquoise (50–60) to dark blue (90–100).

Regarding the social norm, participants in the relevant conditions were given the following information^43,44^: “It is widely accepted that spreading misinformation is wrong and can have a variety of negative outcomes for both individuals and societies. Indeed, a recent study found that more than 80% of U.S. adults think it’s very important to only share accurate content online.” This message thus combined descriptive and injunctive norm elements^29,41,42^.

#### Dynamic changes

Depending on participant choices, their follower count and credibility score changed dynamically. Changes were determined in a probabilistic fashion (see Table 2 for parameters of the normal distributions from which changes were sampled; note that the simulation always rounded displayed change values to integers; descriptive statistics per condition are displayed in Table S2). Engagements with true versus false posts had the same (average) impact on follower numbers except that flagging of false posts was more likely to result in a follower increase than flagging of true posts. Credibility changes were symmetrical, meaning that for true posts, positive engagements (likes, shares) on average improved credibility and negative engagements (flagging) decreased credibility, and vice versa for false posts.

**Table 2:** Parameters determining changes to follower counts and credibility scores.

	Changes to Followers						Changes to Credibility					
	Likes		Shares		Flags		Likes		Shares		Flags	
	*M*	*SD*	*M*	*SD*	*M*	*SD*	*M*	*SD*	*M*	*SD*	*M*	*SD*
True Posts	1	0.75	2	1	0	0.25	0.5	0.5	1	0.5	− 1.5	0.5
False Posts	1	0.75	2	1	0.5	0.25	-0.5	0.5	− 1	0.5	1.5	0.5

### Procedure

Participants initially received an ethics-approved information sheet and provided informed consent and basic demographic information. They were then given task instructions; instructions mentioned the presence of comments, and included information on the source-credibility badge and the social norm in the relevant conditions. Participants then started the social-media simulation, which began with a prompt to “Engage as you would on social media and try to maximize your follower count!”. All participants initially had a follower count of zero. In source-credibility conditions, participants’ initial credibility was set to 50. Participants were then presented with all claims on separate pages and in a random order. For each post, participants decided whether to “like” it, “share” it, or “flag” it (as misleading); they were also able to skip posts, which had no impact on follower count or credibility score. Comments could also be added or liked if desired; this also had no impact on follower number or credibility score. Participants were only able to continue to the next post after 3 s. After the simulation, all claims were presented again, in a plain written format with no images or social-media context features, and belief in each claim was measured on a 0–10 rating scale (from “Certainly false” to “Certainly true”); minimum display time was again 3 s per claim. Participants were then asked whether they could generally see the comments below each post (without scrolling down). Only approximately 7% of participants indicated they did not see the comments; see the supplementary information for results with these participants excluded (Table S3). Finally, participants were asked whether they believed their data should be excluded due to low effort before being fully debriefed. The debriefing sheet included information on why participants were exposed to false claims, and contained a link to a spreadsheet that listed all claims and indicated which were true and which were false^45^. The experiment took approximately 25 min to complete; participants were compensated with GBP 3.75 (approx. USD 4.25).

## Results

Analyses reported below were preregistered at https://osf.io/y4kj5 unless stated otherwise. This study had two between-subjects factors: credibility badge (no badge, badge) and social norm (no norm, norm). Each dependent variable was analyzed using a 2 × 2 between-subjects ANOVA with credibility badge, social norm, and their interaction as predictors. The only exception was the analysis of final achieved credibility; because participants only received credibility scores when credibility badges were present, achieved credibility was analyzed using an independent samples *t*-test with social norm as the between-subjects factor. All analyses were conducted in R statistical software version 4.3.0^46^ and analysis scripts are available at https://osf.io/rbse8/. ANOVAs were conducted using the *aov_ez* function from the package *afex*^47^, follow up t-tests were conducted using the package *rstatix*^48^, assumption checks were conducted using *performance*^49^, and plots were created using *ggplot2*^50^. For all reported analyses, the assumption of homoscedasticity was met (*p* ≥ 0.077) and no outliers were detected using Cook’s distance (all Cook’s D ≤ 0.02). Additionally, visual inspection of quantile–quantile plots revealed the residuals were sufficiently normal to justify conducting parametric analyses, particularly given the relatively large sample size (see^51^ for more details).

### Engagement with social-media posts

**Figure 3:**
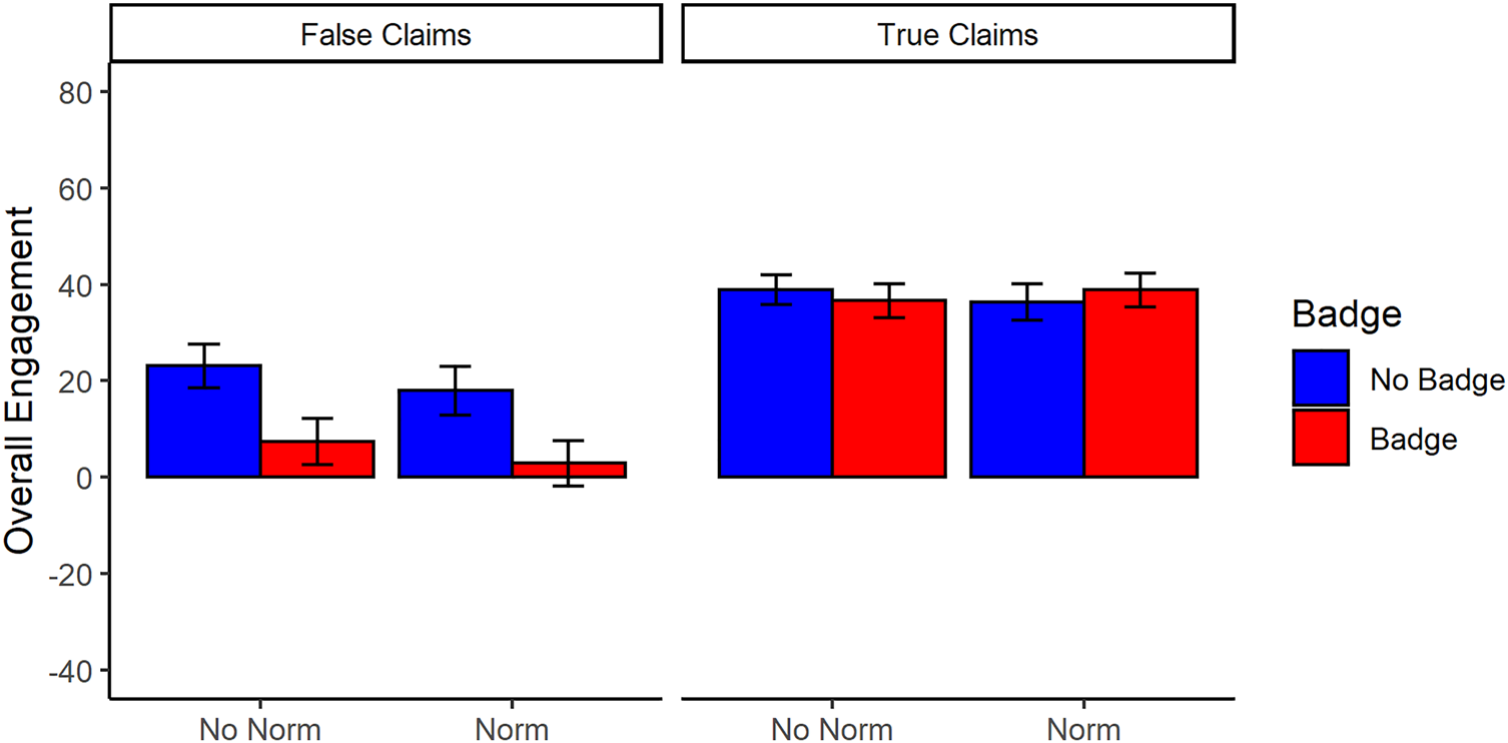
Engagement with posts containing true and false claims for each condition. Error bars represent 95% CIs. Composite scores were calculated by summing the following values for each engagement type across all posts: flag = − 1; skip = 0; like =  + 1; share =  + 2. The possible range of the overall engagement score was thus -40 to 80, with 0 representing a no-engagement baseline.

**Figure 4:**
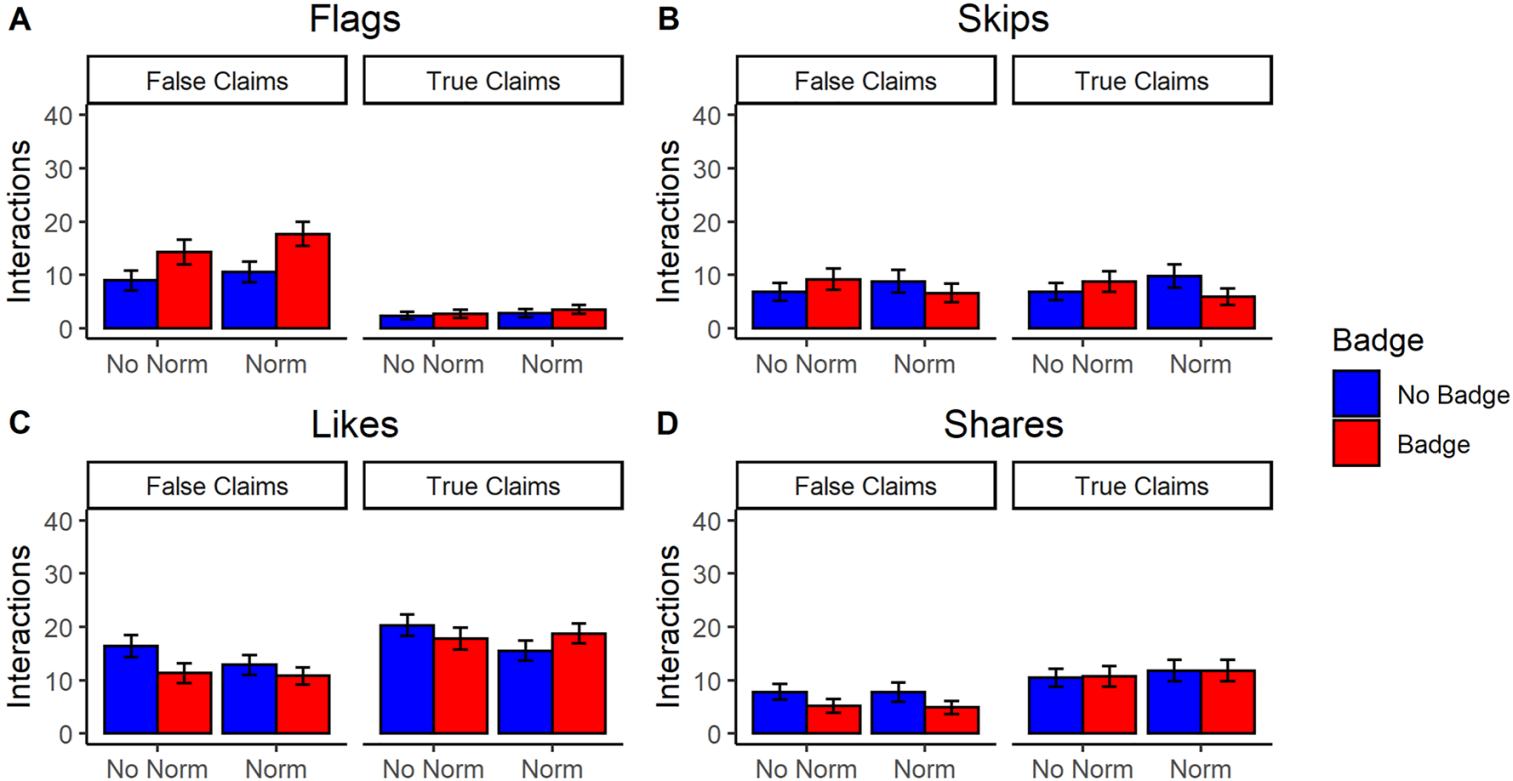
Specific engagements with posts containing true and false claims for each condition. Error bars represent 95% CIs.

Each participant’s engagement with the various posts was first condensed into compound scores for true and false claims. To this end, responses to each post was coded using the following values: flag = − 1; skip = 0; like =  + 1; and share =  + 2 (following^8^). Additional analyses using cumulative-link mixed-effects modelling can be found in the supplementary information. Results are shown in Fig. 3. For true claims, there were no significant main effects of credibility badge or social norm, and no significant interaction, *F*s ≤ 1.91, *p*s ≥ 0.17, η_p_^2^ ≤ 0.005. For false claims, there were significant main effects of credibility badge, *F*(1, 411) = 40.51, *p* < 0.001, η_p_^2^ = 0.09, 95% CI [0.04, 0.15], and social norm, *F*(1, 411) = 4.08, *p* = 0.044, η_p_^2^ = 0.01, 95% CI [0.00, 0.04], with less positive engagements (i.e., less liking/sharing) when credibility badges and a social norm were included, respectively. There was no significant interaction between credibility badge and social norm, *F*(1,411) = 0.02, *p* = 0.898, η_p_^2^ < 0.001, 95% CI [0.00, 0.01]. Figure 4 shows each engagement type (i.e., flag, skip, like, share) graphed separately (see supplementary information for full analyses separated by engagement type).

Finally, we also analyzed whether credibility badges and social norms impacted the final follower counts of participants and their final achieved credibility (the latter only for the credibility-badge conditions). For final follower counts, there were no significant main effects of credibility badge or social norm, and no significant interaction**,** *F*s ≤ 3.30, *p*s ≥ 0.070, η_p_^2^ ≤ 0.008. Additionally, the presence of a social norm did not significantly impact the final credibility achieved by participants, *t*(206) = 1.51, *p* = 0.133, *d* = 0.21.

### Belief

**Figure 5:**
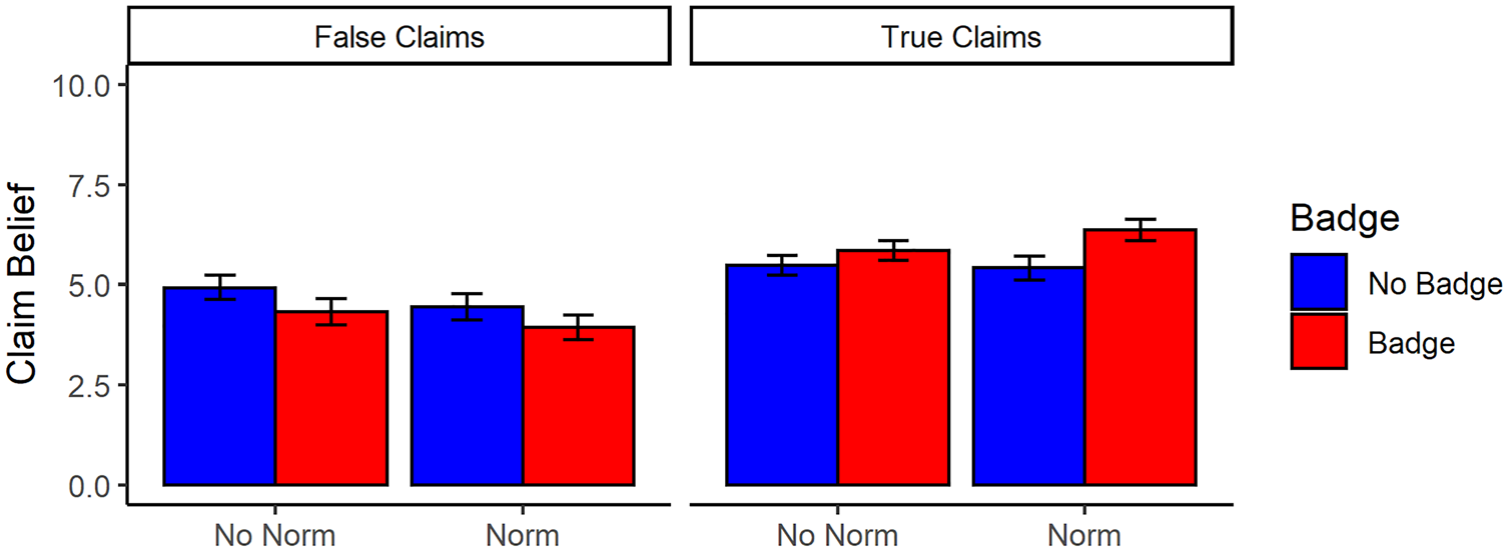
Level of belief in true and false claims for each condition. Errors bars represent 95% CIs.

Beliefs in true and false claims were analyzed in separate ANOVAs (see Fig. 5). For false claims, we found that the presence of a credibility badge led to significantly reduced belief, *F*(1, 411) = 12.34*, p* < 0.001, η_p_^2^ = 0.03, 95% CI [0.01, 0.07]. The social-norm intervention also significantly reduced belief in false claims, *F*(1, 411) = 7.37*, p* = 0.007, η_p_^2^ = 0.02, 95% CI [0.00, 0.05]. However, the interaction between credibility badge and social norm was not significant, *F*(1, 411) = 0.08, *p* = 0.780, η_p_^2^ < 0.001, 95% CI [0.00, 0.01]. For true claims, we found that the presence of a credibility badge led to significantly greater belief than when there was no credibility badge, *F*(1, 411) = 23.89*, p* < 0.001, η_p_^2^ = 0.05, 95% CI [0.02, 0.10]. There was no significant main effect of social norm, *F*(1, 411) = 2.91*, p* = 0.089, η_p_^2^ = 0.007, 95% CI [0.00, 0.03]. However, there was also a significant credibility badge by social norm interaction, *F*(1, 411) = 4.79*, p* = 0.029, η_p_^2^ = 0.01, 95% CI [0.00, 0.04]. Follow-up independent sample *t*-tests revealed that the credibility-badge intervention significantly increased belief in true claims in both the social-norm, *t*(202) = 4,67, *p* < 0.001, *d* = 0.65, 95% CI [0.39, 0.93], and no-social-norm conditions, *t*(205) = 2.07, *p* = 0.040, *d* = 0.29, 95% CI [0.04, 0.58]. However, the interaction occurred because the effect of credibility badges was stronger when a social norm was given.

**Figure 6:**
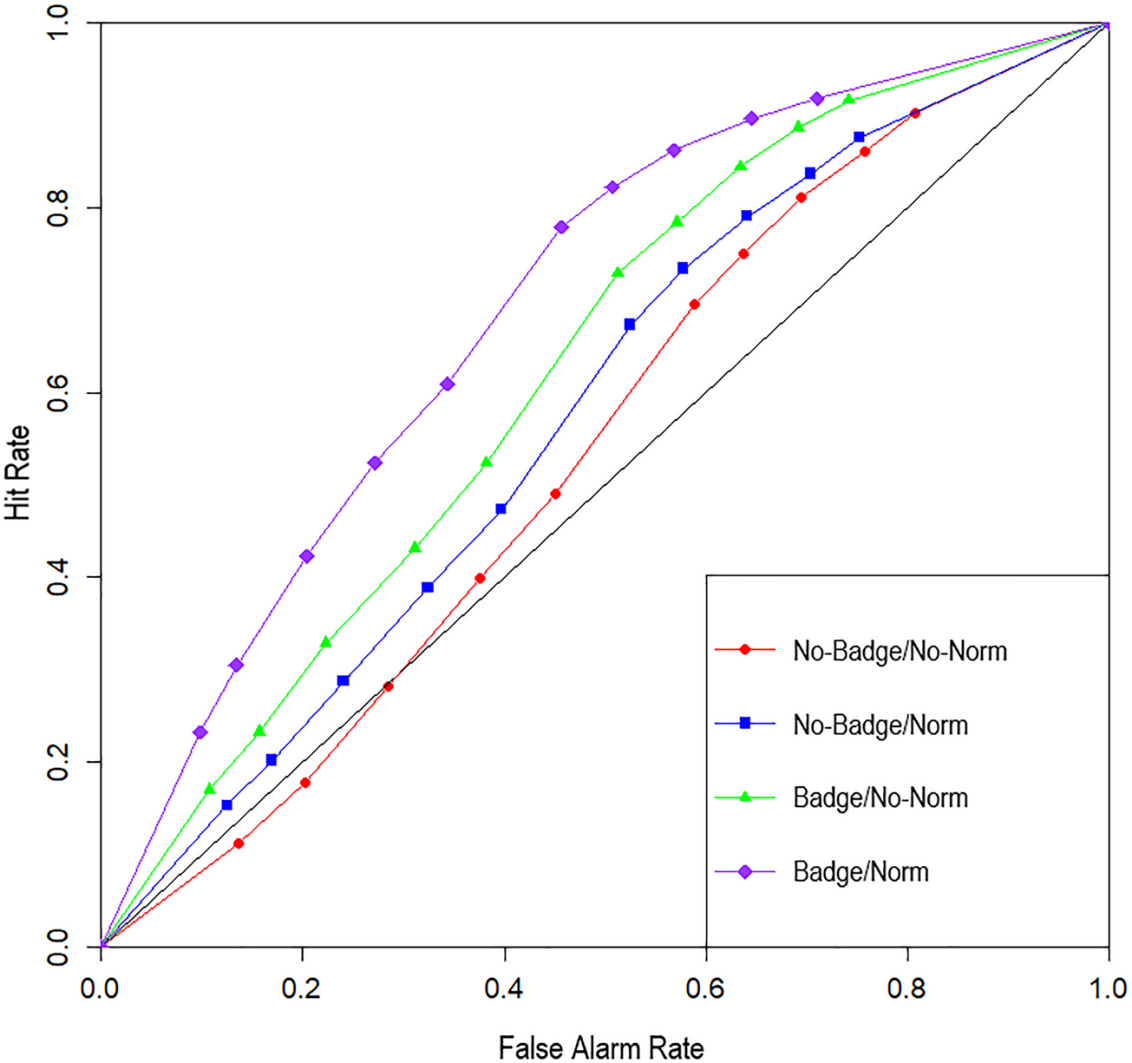
Receiver operating characteristic curves for each condition. Hit rates plotted against false alarm rates at each level of the belief scale (0–10). The diagonal line represents an AUC of 0.5, which indicates chance performance (i.e., no discrimination ability). Hit rates are the proportion of true claims with belief scores ≥ the respective level of the belief scale, and false alarm rates are the analogous proportion for false claims. Note that the highest scale values (i.e., belief score of 10) are displayed in the bottom-left portion of the curves (e.g., in the badge/norm condition, approx. 23% of true claims received a score of 10, but only approx. 8% of false claims); moving rightward along the curves, each successive point represents the next lowest belief-scale value. The point at which the curves all meet at the top right corner can thus be interpreted as each curve’s 11^th^ data point (i.e., 100% of claims had belief ratings ≥ 0).

The effectiveness of the interventions was additionally examined using signal detection theory (for detailed discussions of applying signal detection theory to the study of fake news and misinformation, see^52–54^). Specifically, we used receiver operating characteristic (ROC) analysis^55^, which enables differentiation of discrimination ability (i.e., ability to distinguish between true and false claims) from response bias (i.e., the general tendency to rate claims as true or false). To construct ROC curves, we calculated hit and false alarm rates for each individual participant at each level of the belief measure (i.e., from 0 to 10; see Fig. 6). The plot reflects cumulative rates, that is, each level of the belief measure was treated as a cut-off point^56^. For example, if 5 is the cut-off point, then any true claim rated at 5 or above would be classified as a hit and any false claim rated 5 or above would be classified as a false alarm. In this way, ten hit rates and ten alarm rates were calculated for each participant. We then used the trapezoidal rule^57^ to calculate area under the curve (AUC) for each participant. For AUC, 0.5 represents chance performance (i.e., no ability to discriminate between true and false claims) and 1 represents perfect discrimination ability (i.e., classifying all true claims as true and all false claims as false).

The analysis of discrimination ability (as indexed by AUC) revealed significant main effects of both credibility badge,* F*(1, 411) = 28.95, *p* < 0.001, η_p_^2^ = 0.07, 95% CI [0.03, 0.12], and social norm, *F*(1, 411) = 8.13, *p* = 0.005, η_p_^2^ = 0.02., 95% CI [0.00, 0.05]. However, there was no significant credibility badge by social norm interaction, *F*(1, 411) = 1.29, *p* = 0.256, η_p_^2^ = 0.003. Thus, participants were better able to discriminate between true and false headlines when credibility badges were present and when the social norm was presented.

## Discussion

Cumulatively, the results suggest that credibility badges and social norms can be effective interventions for counteracting online misinformation. However, credibility badges appear to be the more promising of the two interventions. Credibility badges were associated with larger effect sizes and more consistent results across the alternative analysis specifications (see supplementary information). Additionally, only the credibility-badge intervention significantly reduced the sharing of false claims. Incorporating such interventions into social-media platforms may have considerable benefits for the information ecosystem, reducing the extent to which misinformation is believed, amplified, and spread, thereby shifting the overall information balance in favor of more reliable information. Even if such shifts are small, they may be useful given the need for social coordination and a shared factual understanding to tackle public-health challenges like vaccine uptake and sociopolitical challenges like climate action^58^.

Our findings for belief were also promising. Specifically, the presence of credibility badges led to participants having greater belief in true claims and lower belief in false claims. For true beliefs, credibility badges also interacted with the social-norm intervention, with the presence of a social norm enhancing the effectiveness of the credibility-badge intervention (although this interaction was not robust to changes in exclusion criteria, see supplementary information). In part, the effect of credibility badges may have been driven by the relationship between source-credibility scores and post veracity; however, this relationship was arguably too weak to fully explain the results, and as such it is likely that presence of the credibility badges led participants to more deeply consider the veracity of each post, in a manner similar to accuracy nudges^10,43^. The social-norm intervention did not significantly influence belief for true claims, but it did reduce belief in false claims. Additionally, both credibility badges and social norms led to better discernment between true and false claims, that is, both interventions enhanced discernment (see^53,54^). Given the potential negative impacts of misinformation^1,3,8^, these are promising findings because they suggest that these interventions can help people form more accurate beliefs and avoid falling for misinformation online.

Although this study demonstrates that credibility badges may be a useful intervention for reducing misinformation belief and engagement, there are several potential challenges with implementation. For one, within the study all claims came with accurate fact-checks (i.e., corrections of false information, and affirmations of true information). As such, in situations where corrective information is not provided, the effectiveness of these interventions may be weakened. Additionally, within a controlled experimental environment, researchers have a priori knowledge about which claims are true and false. However, in the real world assessing the truthfulness of claims is a difficult and resource-intensive task, and many claims are not amenable to fact-checking^59^. Due to these difficulties, the objective veracity of most claims on social media is unknown or indeterminable. It follows that for most content engagements (e.g., liking, sharing, flagging, etc.), it may not be possible to objectively determine the appropriate impact on the user’s credibility, which may limit the real-world applicability of the intervention. However, even if only a subset of engagements were to impact credibility, the intervention may still be effective. Moreover, even though a claim may not have been fact-checked at the time a user interacts with it, the user’s credibility could still be updated retrospectively (accompanied by feedback) if they had interacted with a post that was later fact-checked. The mere knowledge that social-media engagements may subsequently impact perceived credibility, either immediately or at some point in the future, may be sufficient to motivate users to be more careful when sharing or liking information that may be false. Additionally, as mentioned earlier, the presence of a credibility badge in and of itself may serve as an ongoing prompt to consider the accuracy of information prior to liking or sharing it, which previous research has shown to be an effective intervention to reduce misinformation sharing^10,43^.

There are also several ongoing developments that have the potential to greatly increase the proportion of online claims that can be fact-checked, which would have the flow-on effect of increasing the viability and potential impact of credibility badges. One approach is to take advantage of developments in natural language processing and artificial intelligence to automate fact-checking^60,61^. There have also been recent advances in crowd-sourcing of fact-checks, such as the Birdwatch program (now called “Community Notes”) implemented by Twitter/X^62–64^. Both approaches have the potential to increase the proportion of social-media claims that could be used to calculate and update credibility scores. Whereas the main mechanism investigated in this study was the impact of a credibility score on the user’s own reputation, naturally the more reliable a credibility score, the more it could also serve as a guide in a user’s assessment of source credibility on social media.

In contrast to a credibility badge, informing users of a social norm is relatively straightforward and simple to implement—it would only require presenting social-media users with a simple prompt, either when they first log into the site or when they decide to interact with a post. Providing a prompt at first login may improve the accuracy of the beliefs users form during their time on the platform. Providing the prompt at the point of post engagement may, however, prove more beneficial for reducing sharing and liking of false or misleading posts due to the introduced friction^65^ and the increased salience and proximity of the prompt, memory for which will fade over time. Additionally, a social-norm prompt could easily be combined with an accuracy nudge^10,43^, which may have additive effects. On the negative side, frequent prompts may be ignored by users after some time due to habituation^66^, or may negatively affect the user experience. Finally, the fact that much online content is of unclear veracity or not amenable to fact-checks also means that in some situations users may find it difficult to adhere to the social norm even if they intend to (e.g., when they encounter a piece of misinformation that appears plausible to them).

An additional potential limitation of the interventions we propose is that people can have a diverse set of motivations for sharing information online, some of which may be unrelated to the accuracy of the information. For example, people may share misinformation due to identity-performative motivations (e.g., to feel a sense of group belonging or elicit an emotional reaction from others)^67^, or because a piece of news would be interesting-if-true^68^. These motivations may play a greater role in a real-world social-media environment than the confines of a simulation and may be more prevalent for some types of misinformation (e.g., political misinformation). By shifting users’ attention toward the accuracy of the information they are engaging with, the proposed interventions may limit the impact of these other motivations, which may or may not be desirable. Additionally, as credibility badges would draw attention to a user’s regular sharing of false or misleading information regardless of the underlying motivation for doing so, they may be inappropriate in certain cases (e.g., satirical accounts). The credibility-badge intervention would also not be effective, and may even backfire, in cases where a source that is usually credible, such as a reputable news or scientific organization, spreads misinformation. Because the bulk of information put out by these organizations is accurate, and therefore they would be assigned a high credibility rating, it may mean that any misinformation they produce (whether accidental or otherwise) may be more easily believed and more difficult to be corrected. Indeed, if people came to focus solely on overall source credibility, without considering the veracity of the specific claim or news story, that would be an undesirable outcome (although the risk of this seems low given there is evidence that people generally focus more on story plausibility than the source e.g.,^69^). Future research should also examine whether intervention effectiveness is impacted by post characteristics, such as length, language, and visuals (e.g., images, gifs, video etc.), and/or individual characteristics, such as political ideology and trust in institutions.

Overall, the results from this study highlight that both credibility-badge and social-norm interventions have the potential to improve the information landscape on social media^65^. Both a credibility badge and a social norm led to reduced belief in false claims, and improvements in people’s ability to discriminate between true and false information. Additionally, credibility badges improved the way participants engaged with information, leading to increased flagging and decreased liking and sharing of false posts. Our main preregistered analyses also showed that social norms increased flagging and decreased liking of false posts, but these results were less robust. Credibility badges would also have the additional benefit of providing social-media users with usually opaque information about the credibility of other users, allowing them to more easily assess the likelihood that the source is reliable. Cumulatively, the findings of this study suggest that both credibility badges and the provision of social norms are promising potential additions to the growing suite of tools used to fight misinformation online.

### Supplementary Information

Supplementary Information.

## Data Availability

Data and materials from the empirical studies are available at on the Open Science Framework at https://osf.io/rbse8/. The study was preregistered at https://osf.io/y4kj5.

## References

[1] Lewandowsky S, Ecker UKH, Cook J (2017). Beyond misinformation: Understanding and coping with the “post-truth” era. J. Appl. Res. Mem. Cogn..

[2] Loomba S, de Figueiredo A, Piatek SJ, de Graaf K, Larson HJ (2021). Measuring the impact of COVID-19 vaccine misinformation on vaccination intent in the UK and USA. Nat. Hum. Behav..

[3] Swire-Thompson B, Lazer D (2022). Reducing health misinformation in science: A call to arms. Ann. Am. Acad. Pol. Soc. Sci..

[4] Ecker UKH, O’Reilly Z, Reid JS, Chang EP (2020). The effectiveness of short-format refutational fact-checks. Br. J. Psychol..

[5] Guess AM (2020). A digital media literacy intervention increases discernment between mainstream and false news in the United States and India. Proc. Natl. Acad. Sci. U.S.A..

[6] Lobato EJC, Powell M, Padilla LMK, Holbrook C (2020). Factors predicting willingness to share COVID-19 misinformation. Front. Psychol..

[7] Lewandowsky S, van der Linden S (2021). Countering misinformation and fake news through inoculation and prebunking. Eur. Rev. Soc. Psychol..

[8] MacFarlane D, Tay LQ, Hurlstone MJ, Ecker UKH (2021). Refuting spurious COVID-19 treatment claims reduces demand and misinformation sharing. J. Appl. Res. Mem. Cogn..

[9] Pennycook G, Rand DG (2019). Fighting misinformation on social media using crowdsourced judgments of news source quality. Proc. Natl. Acad. Sci. U.S.A..

[10] Pennycook G, McPhetres J, Zhang Y, Lu JG, Rand DG (2020). Fighting COVID-19 misinformation on social media: Experimental evidence for a scalable accuracy-nudge intervention. Psychol. Sci..

[11] Tay LQ, Hurlstone MJ, Kurz T, Ecker UKH (2022). A comparison of prebunking and debunking interventions for implied versus explicit misinformation. Br. J. Psychol..

[12] Ecker UKH (2022). The psychological drivers of misinformation belief and its resistance to correction. Nat. Rev. Psychol..

[13] Pennycook G, Rand DG (2021). The psychology of fake news. Trends Cogn. Sci..

[14] Briñol P, Petty RE (2009). Source factors in persuasion: A self-validation approach. Eur. Rev. Soc. Psychol..

[15] Pornpitakpan C (2004). The persuasiveness of source credibility: A critical review of five decades’ evidence. J. Appl. Soc. Psychol..

[16] Sparks JR, Rapp DN (2011). Readers’ reliance on source credibility in the service of comprehension. J. Exp. Psychol. Learn. Mem. Cogn..

[17] Chaiken S, Maheswaran D (1994). Heuristic processing can bias systematic processing: Effects of source credibility, argument ambiguity, and task importance on attitude judgment. J. Pers. Soc. Psychol..

[18] Amazeen MA, Krishna A (2023). Processing vaccine misinformation: Recall and effects of source type on claim accuracy via perceived motivations and credibility. Int. J. Commun..

[19] Nadarevic L, Reber R, Helmecke AJ, Köse D (2020). Perceived truth of statements and simulated social media postings: An experimental investigation of source credibility, repeated exposure, and presentation format. Cogn. Res. Princ. Implic..

[20] Walter N, Tukachinsky R (2020). A meta-analytic examination of the continued influence of misinformation in the face of correction: How powerful is it, why does it happen, and how to stop it?. Commun. Res..

[21] Ecker UKH, Antonio LM (2021). Can you believe it? An investigation into the impact of retraction source credibility on the continued influence effect. Mem. Cognit..

[22] Guillory JJ, Geraci L (2013). Correcting erroneous inferences in memory: The role of source credibility. J. Appl. Res. Mem. Cogn..

[23] Vraga EK, Bode L (2018). I do not believe you: How providing a source corrects health misperceptions across social media platforms. Inf. Commun. Soc..

[24] Kim A, Moravec PL, Dennis AR (2019). Combating fake news on social media with source ratings: The effects of user and expert reputation ratings. J. Manag. Inf. Syst..

[25] Cottrell CA, Neuberg SL, Li NP (2007). What do people desire in others? A sociofunctional perspective on the importance of different valued characteristics. J. Pers. Soc. Psychol..

[26] Altay S, Hacquin A-S, Mercier H (2022). Why do so few people share fake news? It hurts their reputation. New Media Soc..

[27] Nyhan B, Reifler J (2015). The effect of fact-checking on elites: A field experiment on U.S. state legislators. Am. J. Polit. Sci..

[28] Brown GDA, Lewandowsky S, Huang Z (2022). Social sampling and expressed attitudes: Authenticity preference and social extremeness aversion lead to social norm effects and polarization. Psychol. Rev..

[29] Cialdini RB (2003). Crafting normative messages to protect the environment. Curr. Dir. Psychol. Sci..

[30] Hornsey MJ, Fielding KS (2017). Attitude roots and Jiu Jitsu persuasion: Understanding and overcoming the motivated rejection of science. Am. Psychol..

[31] Kaplan MF, Miller CE (1987). Group decision making and normative versus informational influence: Effects of type of issue and assigned decision rule. J. Pers. Soc. Psychol..

[32] Avram M, Micallef N, Patil S, Menczer F (2020). Exposure to social engagement metrics increases vulnerability to misinformation. Harv. Kennedy Sch. Misinformation Rev..

[33] Butler LH, Fay N, Ecker UKH (2023). Social endorsement influences the continued belief in corrected misinformation. J. Appl. Res. Mem. Cogn..

[34] Vlasceanu M, Coman A (2022). The impact of social norms on health-related belief update. Appl. Psychol. Health Well-Being.

[35] Ecker UKH (2022). Combining refutations and social norms increases belief change. Q. J. Exp. Psychol..

[36] Andı S, Akesson J (2020). Nudging away false news: Evidence from a social norms experiment. Digit. Journal..

[37] Gimpel H, Heger S, Olenberger C, Utz L (2021). The effectiveness of social norms in fighting fake news on social media. J. Manag. Inf. Syst..

[38] Epstein Z (2021). Developing an accuracy-prompt toolkit to reduce COVID-19 misinformation online. Harv. Kennedy Sch. HKS Misinformation Rev..

[39] Jones CM (2023). Impact of social reference cues on misinformation sharing on social media: Series of experimental studies. J. Med. Internet Res..

[40] Butler LH (2023). The (Mis)Information Game: A social media simulator. Behav. Res. Methods.

[41] Hamann KRS, Reese G, Seewald D, Loeschinger DC (2015). Affixing the theory of normative conduct (to your mailbox): Injunctive and descriptive norms as predictors of anti-ads sticker use. J. Environ. Psychol..

[42] Smith JR, Louis WR (2008). Do as we say and as we do: The interplay of descriptive and injunctive group norms in the attitude–behaviour relationship. Br. J. Soc. Psychol..

[43] Pennycook G (2021). Shifting attention to accuracy can reduce misinformation online. Nature.

[44] Poushter, J., Fagan, M. & Gubbala, S. *Climate change remains top global threat across 19-country survey*. (2022).

[45] Greene CM (2022). Best practices for ethical conduct of misinformation research: A scoping review and critical commentary. Eur. Psychol..

[46] R Core Team. *R: A Language and Environment for Statistical Computing*. (R Foundation for Statistical Computing, 2023).

[47] Singmann, H., Bolker, B., Westfall, J., Aust, F. & Ben-Shachar, M. S. *afex: Analysis of Factorial Experiments*. (2023).

[48] Kassambara, A. *rstatix: Pipe-Friendly Framework for Basic Statistical Tests*. (2023).

[49] Lüdecke D, Ben-Shachar MS, Patil I, Waggoner P, Makowski D (2021). performance: An R package for assessment, comparison and testing of statistical models. J. Open Source Softw..

[50] Wickham H (2016). ggplot2: Elegant Graphics for Data Analysis.

[51] Lumley T, Diehr P, Emerson S, Chen L (2002). The importance of the normality assumption in large public health data sets. Annu. Rev. Public Health.

[52] Batailler C, Brannon SM, Teas PE, Gawronski B (2022). A signal detection approach to understanding the identification of fake news. Perspect. Psychol. Sci..

[53] Modirrousta-Galian A, Higham PA (2023). Gamified inoculation interventions do not improve discrimination between true and fake news: Reanalyzing existing research with receiver operating characteristic analysis. J. Exp. Psychol. Gen..

[54] Guay B, Berinsky AJ, Pennycook G, Rand D (2023). How to think about whether misinformation interventions work. Nat. Hum. Behav..

[55] Higham PA, Higham DP (2019). New improved gamma: Enhancing the accuracy of Goodman–Kruskal’s gamma using ROC curves. Behav. Res. Methods.

[56] Mandrekar JN (2010). Receiver operating characteristic curve in diagnostic test assessment. J. Thorac. Oncol..

[57] Pollack I, Hsieh R (1969). Sampling variability of the area under the ROC-curve and of d’e. Psychol. Bull..

[58] Van Lange PAM, Rand DG (2022). Human cooperation and the crises of climate change, COVID-19, and misinformation. Annu. Rev. Psychol..

[59] Arnold, P. *The challenges of online fact checking*. https://fullfact.org/blog/2020/dec/the-challenges-of-online-fact-checking-how-technology-can-and-cant-help/ (2020).

[60] Zeng X, Abumansour AS, Zubiaga A (2021). Automated fact-checking: A survey. Lang. Linguist. Compass.

[61] Liu X, Nielek R, Adamska P, Wierzbicki A, Aberer K (2015). Towards a highly effective and robust Web credibility evaluation system. Decis. Support Syst..

[62] Pröllochs, N. Community-based fact-checking on twitter’s birdwatch platform. 10.48550/arXiv.2104.07175 (2021).

[63] Saeed, M., Traub, N., Nicolas, M., Demartini, G. & Papotti, P. Crowdsourced fact-checking at twitter: How does the crowd compare with experts? In *Proceedings of the 31st ACM International Conference on Information & Knowledge Management* 1736–1746 10.1145/3511808.3557279 (ACM, 2022).

[64] Allen, J., Martel, C. & Rand, D. G. Birds of a feather don’t fact-check each other: Partisanship and the evaluation of news in Twitter’s Birdwatch crowdsourced fact-checking program. In *CHI Conference on Human Factors in Computing Systems* 1–19 10.1145/3491102.3502040 (ACM, 2022).

[65] Kozyreva A, Lewandowsky S, Hertwig R (2020). Citizens versus the internet: Confronting digital challenges with cognitive tools. Psychol. Sci. Public Interest.

[66] Anderson BB, Jenkins JL, Vance A, Kirwan CB, Eargle D (2016). Your memory is working against you: How eye tracking and memory explain habituation to security warnings. Decis. Support Syst..

[67] Chadwick A, Vaccari C, Kaiser J (2022). The amplification of exaggerated and false news on social media: The roles of platform use, motivations, affect, and ideology. Am. Behav. Sci..

[68] Altay S, de Araujo E, Mercier H (2022). “If this account is true, it is most enormously wonderful”: Interestingness-if-true and the sharing of true and false news. Digit. J..

[69] Dias N, Pennycook G, Rand DG (2020). Emphasizing publishers does not effectively reduce susceptibility to misinformation on social media. Harv. Kennedy Sch. HKS Misinformation Rev..

