# Supplementary material for: Source-credibility information and social norms improve truth discernment and reduce engagement with misinformation online
Source: Sci Rep. 2024 Mar 22;14:6900. doi: 10.1038/s41598-024-57560-7 (PMC10960008; doi:10.1038/s41598-024-57560-7)
Supplement: Supplementary file 1 — Supplementary Information. [file 41598_2024_57560_MOESM1_ESM.docx]

# ­ Source-Credibility Information and Social Norms Improve Truth Discernment and Reduce Engagement with Misinformation Online

# Supplementary Information

**Table S1**

Number of displayed post likes, shares, and flags in each condition. Due to the zero-truncation, these values differed from the parameters

used to determine the number of likes, shares, and flags in a systematic and predictable way. Specifically, for all conditions the actual means were higher than the parameterized means, whereas the actual standard deviations were lower. This is because the parameterized means were relatively close to zero, and the standard deviations were large. This led to many values being truncated at zero, whereas in the opposite direction numbers were able to be much larger than the parameterized means.

|  |  | Likes | | Shares | | Flags | |
| --- | --- | --- | --- | --- | --- | --- | --- |
| Condition |  | *M* | *SD* | *M* | *SD* | *M* | *SD* |
| Badge/Norm | True Posts | 10.04 | 6.84 | 2.80 | 2.04 | 0.05 | 0.21 |
|  | False Posts | 10.07 | 6.96 | 2.79 | 2.02 | 1.00 | 0.80 |
| Badge/No-Norm | True Posts | 10.28 | 7.07 | 2.77 | 2.04 | 0.05 | 0.21 |
|  | False Posts | 10.22 | 7.00 | 2.75 | 2.00 | 0.98 | 0.79 |
| No-Badge/Norm | True Posts | 9.99 | 7.04 | 2.77 | 2.04 | 0.04 | 0.20 |
|  | False Posts | 10.06 | 6.95 | 2.82 | 2.05 | 0.98 | 0.77 |
| No-Badge/No-Norm | True Posts | 10.15 | 7.03 | 2.82 | 2.06 | 0.05 | 0.21 |
|  | False Posts | 10.13 | 6.96 | 2.81 | 2.03 | 0.99 | 0.78 |

**Table S2**

Changes to followers and credibility in each condition.

|  |  | Changes to Followers | | | | | |  | Changes to Credibility | | | | | |
| --- | --- | --- | --- | --- | --- | --- | --- | --- | --- | --- | --- | --- | --- | --- |
|  |  | Likes | | Shares | | Flags | |  | Likes | | Shares | | Flags | |
| Condition |  | *M* | *SD* | *M* | *SD* | *M* | *SD* |  | *M* | *SD* | *M* | *SD* | *M* | *SD* |
| Badge/Norm | True Posts | 1.00 | 0.86 | 2.03 | 1.09 | -0.01 | 0.47 |  | 0.47 | 0.66 | 0.87 | 0.68 | -1.51 | 0.61 |
|  | False Posts | 0.96 | 0.83 | 1.92 | 1.10 | 0.50 | 0.51 |  | -0.49 | 0.62 | -1.04 | 0.65 | 1.32 | 0.77 |
| Badge/No-Norm | True Posts | 1.01 | 0.85 | 2.08 | 1.08 | 0.00 | 0.44 |  | 0.49 | 0.64 | 0.87 | 0.69 | -1.46 | 0.64 |
|  | False Posts | 0.99 | 0.86 | 2.05 | 1.07 | 0.50 | 0.50 |  | -0.49 | 0.63 | -1.03 | 0.62 | 1.35 | 0.77 |
| No-Badge/Norm | True Posts | 1.01 | 0.85 | 2.02 | 1.11 | 0.07 | 0.43 |  |  |  |  |  |  |  |
|  | False Posts | 0.98 | 0.85 | 2.04 | 1.11 | 0.48 | 0.51 |  |  |  |  |  |  |  |
| No-Badge/No-Norm | True Posts | 1.02 | 0.86 | 1.94 | 1.11 | 0.01 | 0.48 |  |  |  |  |  |  |  |
|  | False Posts | 1.00 | 0.84 | 1.98 | 1.09 | 0.50 | 0.52 |  |  |  |  |  |  |  |

## Detailed Analyses of Engagement with Social-Media Posts

To further investigate how participants interacted with true and false claims specifically, each of the potential post interactions (flags, skips, likes, shares) was analyzed in a separate zero-inflated Poisson regression with credibility badge and social norm as predictors. (Note: Due to excessive zeros for the individual types of post interaction, we opted to deviate from our preregistered analysis plan and use zero-inflated Poisson regression rather than ANOVA.) The zero-inflated Poisson regressions were conducted using the *zeroinfl* function from the R package *pscl* [1,2]. Because we were interested in overall main effects of credibility badge and social norm and the interaction, we conducted analyses of deviance on the fitted models using the Anova function from the *car* package [3].

### Flags

For flags of true claims, there was a marginally significant main effect of social norm, *χ^2^*(1) = 4.95, *p*= .026, with the social norm leading to a slight increase in the flagging of true claims, but there was no main effect of credibility badge and no significant interaction, *χ^2^*(1) ≤ 1.30, *p*≥ .254. For false claims, there were significant main effects of both credibility badge, *χ^2^*(1) = 263.32, *p*< .001, and social norm, *χ^2^*(1) = 18.19, *p*< .001, with both interventions leading to greater flagging of false claims. There was no significant credibility badge by social norm interaction, *χ^2^*(1) = 0.06, *p*= .805.

### Skips

For skips of true claims, there were significant main effects of both credibility badge, *χ^2^*(1) = 12.72, *p*< .001, and social norm, *χ^2^*(1) = 5.45, *p*= .020; however, these were qualified by a significant interaction, *χ^2^*(1) = 62.95, *p*< .001. To further examine this interaction, two separate zero-inflated Poisson regressions were used to analyze the effect of credibility badges when a social norm was and was not also given. These analyses showed that when the social norm was presented, credibility badges led to significantly fewer skips, *χ^2^*(1) = 66.59, *p*< .001; however, when there was no social norm given, credibility badges led to significantly more skips, *χ^2^*(1) = 9.08, *p*= .003. For false claims, there were no significant main effects, *χ^2^*(1) ≤ 0.18, *p*≥ .674; however, there was a significant credibility badge by social norm interaction, *χ^2^*(1) = 21.94, *p*< .001. The same procedure was used to conduct follow-up tests, revealing that when a social norm was presented, credibility badges led to decreased skipping of false posts, *χ^2^*(1) = 12.12, *p*< .001; however, when there was no social norm presented, credibility badges led to increased skipping of false posts, *χ^2^*(1) = 9.85, *p*= .002.

### Likes

Unlike all other post interactions, there were not excesses zeros for likes of true claims. Therefore, analyses for likes of true claims were conducted using a standard Poisson regression. For likes of true claims, there was no significant main effect of credibility badge, *χ^2^*(1) = 0.81, *p*= .369. However, there was a significant main effect of social norm, *χ^2^*(1) = 20.76, *p*< .001, and a significant interaction, *χ^2^*(1) = 46.47, *p*< .001. Follow-up Poisson regressions revealed that when the social norm was presented, credibility badges led to significantly more likes for true claims, *χ^2^*(1) = 30.92, *p*< .001; however, when there was no social norm presented, credibility badges led to fewer likes, *χ^2^*(1) = 16.36, *p*< .001. False claims were again analyzed using zero-inflated Poisson regression. For false claims, there were significant main effects of both credibility badge, *χ^2^*(1) = 78.61, *p*< .001, and social norm, *χ^2^*(1) = 26.82, *p*< .001, with both interventions leading to fewer likes. However, these main effects were qualified by a significant interaction, *χ^2^*(1) = 5.72, *p*= .017. Follow-up zero-inflated Poisson regressions revealed that credibility badges led to reduced liking of false posts both when a social norm was presented, *χ^2^*(1) = 18.71, *p*< .001, and when it was not, *χ^2^*(1) = 65.63, *p*< .001; however, the interaction occurred because the effect of credibility badges on liking was larger when the social norm was not presented.

### Shares

For sharing of true claims, there was a significant main effect of social norm, *χ^2^*(1) = 28.69, *p*< .001, with the social norm leading to increased sharing of true claims. There was no significant main effect of credibility badge nor an interaction, *χ^2^*(1) ≤ 1.76, *p*≥ .185. For false claims, there was a significant main effect of credibility badge, *χ^2^*(1) = 80.34, *p*< .001 and a significant interaction, *χ^2^*(1) = 9.48, *p*= .002, but no significant main effect of social norm, *χ^2^*(1) = 2.26, *p*= .133. Follow-up zero-inflated Poisson regressions revealed that credibility badges led to decreased sharing of false posts both when a social norm was presented, *χ^2^*(1) = 71.81, *p*< .001, and when it was not, *χ^2^*(1) = 18.00, *p*< .001; however, the interaction occurred because the effect of credibility badges was larger when the social norm was also presented.

## Cumulative-Link Mixed-Effects Modelling Results

Given post engagement was technically measured on a 4-point ordinal scale (with levels -1 [flag], 0 [skip], 1 [like], and 2 [share]), engagement with true and false claims was additionally analyzed using cumulative-link mixed-effects modelling (CLMM; see [4]). All analyses were conducted using the *clmm* function of the *ordinal* R package [5]. We then used the *Anova.clmm* function of the R package *RVAideMemoire* [6] to test for overall main effects (rather than effects relative to the reference group). Prior to analysis, credibility badge (no badge; badge) and social norm (no norm, norm) were centered and the dependent variable of reaction type was coded as an ordinal factor with 4 levels (-1–2). Results were analyzed using the model: Reaction Type ~ Credibility Badge × Social Norm + (1 | Participant ID) + (1 + Number of Post Likes + Number of Post Shares + Number of Post Flags | Post ID) + (1 | Source ID). For false claims, there was a significant main effect of credibility badge, *χ^2^*(1) = 35.84, *p*< .001 with less positive engagements (i.e., liking/sharing) when credibility badges were included. There was no statistical evidence of an effect of social-norm inclusion, *χ^2^*(1) = 0.57, *p*≥ .999, or an interaction between credibility badge and social norm, *χ^2^*(1) = 5.75, *p*≥ .999. For true claims, there was no significant effect of credibility badges, *χ^2^*(1) = 0.03, *p*= .874, social norms, *χ^2^*(1) = 0.18, *p*= .675, or their interaction, *χ^2^*(1) = 0.94, *p*≥ .999, on post engagement.

## Excluding Participants Who Could Not Usually See Comments

Because fact-checks for the posts were presented via comments underneath each post, we preregistered supplementary analyses comparing those who participants who did versus did not indicate they had generally seen the comments. While the vast majority of participants reported that they could generally see the comments (93.2%), we repeated the main analyses excluding those participants who reported they generally could not. Table S3 summarizes all results that changed from significant to nonsignificant (or vice versa) based on these exclusions.

**Table S3**

Differences in results based on exclusions.

| Dependent Variable | Effect | With Comment Exclusions | All Participants Included |
| --- | --- | --- | --- |
| False Claims, Compound Social-Media Engagement | Social Norm | *F*(1, 383) = 2.54, *p* = .112, η_p_^2^ = .01, 95% CI [.00, .03] | *F*(1, 411) = 4.08, *p* = .044, η_p_^2^ = .01, 95% CI [.00, .04] |
| False Claims, Share | Social Norm | *χ^2^*(1) = 5.14, *p*= .023 | *χ^2^*(1) = 2.26, *p*= .133 |
| True Claims, Flags | Social Norm | *χ^2^*(1) = 2.87, *p*= .090 | *χ^2^*(1) = 4.95, *p*= .026 |
| True Claims,  Skips | Credibility Badge in No Social Norm Condition | *χ^2^*(1) = 3.63, *p*= .057 | *χ^2^*(1) = 9.08, *p*= .003 |
| True Claims, Shares | Credibility Badge * Social Norm | *χ^2^*(1) = 7.30, *p*= .007 | *χ^2^*(1) = 1.76, *p*= .185 |
| True Claims, Belief | Credibility Badge * Social Norm | *F*(1, 383) = 2.38, *p* = .124, η_p_^2^ = .01, 95% CI [.00, .03] | *F*(1, 411) = 4.79*, p* = .029, η_p_^2^ = .01, 95% CI [.00, .04] |

# References

1. Jackman, S. *pscl: Classes and methods for R developed in the Political Science Computational Laboratory*. (United States Studies Centre, University of Sydney, 2020).

2. Zeileis, A., Kleiber, C. & Jackman, S. Regression models for count data in R. *J. Stat. Softw.* **27**, (2008).

3. Fox, J. & Weisberg, S. *An R Companion to Applied Regression*. (Sage, 2019).

4. McElreath, R. *Statistical rethinking: A Bayesian course with examples in R and Stan*. https://doi.org/10.1201/9781315372495 (CRC Press, 2020).

5. Christensen, R. H. B. *ordinal—Regression Models for Ordinal Data.* (2022).

6. Herve, M. *RVAideMemoire: Testing and Plotting Procedures for Biostatistics*. (2023).
